# Facteurs pronostiques de mortalité par accident vasculaire cérébral artériel à la phase aiguë dans une population nord-africaine

**DOI:** 10.11604/pamj.2020.35.50.16287

**Published:** 2020-02-20

**Authors:** Khadija Sonda Moalla, Mariem Damak, Olfa Chakroun, Nouha Farhat, Salma Sakka, Olfa Hdiji, Hanen Haj Kacem, Noureddine Rekik, Chokri Mhiri

**Affiliations:** 1 Service de Neurologie, Hôpital Universitaire Habib Bourguiba, Sfax, Tunisie; 2 Service des Urgences et du SAMU, Hôpital Universitaire Habib Bourguiba, Sfax, Tunisie

**Keywords:** Accident vasculaire cérébral, mortalité, pronostic, Stroke, mortality, prognosis

## Abstract

**Introduction:**

l’accident vasculaire cérébral (AVC) constitue un problème majeur de santé publique, tant par le nombre de personnes atteintes, que par ses conséquences médicales, sociales et économiques. L’objectif était de dégager les facteurs de mauvais pronostic vital à la phase aiguë de l’AVC artériel.

**Méthodes:**

il s’agit d’une étude prospective durant quatre mois portant sur les patients présentant une symptomatologie évocatrice d’AVC aux deux CHU de Sfax, Tunisie. Le suivi a été d’un mois.

**Résultats:**

nous avons colligé 200 patients. Après un mois de suivi, la mortalité était de 19,9%. Les facteurs de mauvais pronostic vital étaient: le sexe masculin, la consommation de tabac, l’antécédent d’AVC, le score de Glasgow bas, le NIHSS élevé, les céphalées, les crises épileptiques symptomatiques aiguës, le signe de Babinski, la mydriase, l’aphasie, la déviation conjuguée de la tête et des yeux, les chiffres élevés de pression artérielle systolique (PAS), pression artérielle diastolique (PAD) et pression artérielle pulmonaire (PAP), l’hyperthermie, l’hyperglycémie, l’hyperleucocytose, l’augmentation des CRP, créatinine, urée et la troponine Tc, la nature hémorragique de l’AVC, l’œdème péri lésionnel, l’effet de masse, l’engagement, la topographie sylvienne totale de l’ischémie, la présence de signes précoces d’ischémie, l’hémorragie méningée, l’inondation ventriculaire, l’hydrocéphalie, le recours à une assistance respiratoire, au traitement anti-œdémateux et antihypertenseur, la transformation hémorragique, l’épilepsie vasculaire, les complications infectieuses, métaboliques et de décubitus.

**Conclusion:**

l’identification des facteurs prédictifs du devenir vital permet d’optimiser les procédures thérapeutiques et mieux organiser les filières de prise en charge. Une étude comparative sera envisagée afin de mesurer l’impact des mesures correctives.

## Abstract (en)

**Introduction:**

cerebrovascular accident (stroke) constitutes a major public health problem due to the number of people affected and to its medical social and economic consequences. This study aims to identify poor vital prognostic factors for survival in patients with acute arterial stroke.

**Methods:**

we conducted a prospective study of patients with symptoms suggestive of stroke at the two University Hospitals of Sfax, Tunisia over a period of 4 months. Patients were followed-up for a period of 1 month.

**Results:**

we collected data from 200 patients. After one month of follow-up, mortality was 19.9%. Poor prognostic factors were: male sex, consumption of tobacco, a history of stroke, low Glasgow score, high NIHSS, headaches, acute symptomatic epileptic seizures, Babinski’s sign, mydriasis, aphasia, combined deviation of the head and the eyes, high PAS, PAD and PAM, hyperthermia, hyperglycaemia, leukocytosis, high concentration of CRP, creatinine, urea and troponin T, haemorrhagic stroke, perilesional oedema, a mass effect, commitment, total middle cerebral artery topography of ischemia, early signs of ischemia, meningeal hemorrhage, ventricular flood, hydrocephalus, the recourse to respiratory support, to anti-edematous treatment and to antihypertensive therapy, hemorrhagic transformation, vascular epilepsy, infectious, metabolic complications, complications of bed sores.

**Conclusion:**

the identification of the predictive factors for survival allows for optimisation of therapeutic procedures and better implementation of patient’ management. A comparative study will be considered to measure the impact of the corrective measures.

## Introduction

L’accident vasculaire cérébral (AVC) représente une pathologie fréquente, grave et souvent invalidante. En Tunisie, l’incidence annuelle des AVC est estimée à 192 pour 100 000 habitants [1]. Sa prévalence est de 7,2 pour 1 000 habitants [2]. Les facteurs pronostiques propres à notre population n’ont pas été étudiés. Ce travail vise à dégager les facteurs prédictifs du pronostic vital à la phase aiguë de l’AVC artériel dans une population du sud tunisien.

## Méthodes

Il s’agit d’une étude prospective multicentrique incluant deux centres hospitalo-universitaires de la ville de Sfax. Les inclusions ont eu lieu sur quatre mois et ont concerné les patients ayant présenté ou consulté pour une symptomatologie évocatrice d’un AIT ou d’un AVC selon la définition de l’OMS. Cette symptomatologie devait dater de moins de 72 heures. Le suivi des patients était d’un mois. Nous avons exclu les patients âgés de moins de 18 ans et ceux n’ayant pas eu une imagerie cérébrale (TDM et/ou IRM). Les patients perdus de vue ou dont l’imagerie cérébrale a montré une thrombose veineuse cérébrale ou une hémorragie sous arachnoïdienne sans hématome intra parenchymateux associé ou bien un hématome extra ou sous dural ont été également exclus. Nous avons recueilli les données démographiques, cliniques, biologiques et radiologiques. L’évaluation clinique de tous les patients a été réalisée par le même investigateur. L’état de conscience a été évalué lors du premier examen clinique par le score de Glasgow [3]. L’état neurologique des patients durant le suivi a été évalué par le National Institute of Health Stroke Score ou NIHSS [4]. L’état fonctionnel a été évalué par l’échelle de mesure d’indépendance fonctionnelle ou MIF [5]. Le patient a été considéré comme dépendant si le score MIF est inférieur à 126. Le patient a été considéré comme dépendant si le score MIF est inférieur à 126. Un bilan biologique a été pratiqué dans les premières 72 heures comportant: numération formule sanguine (NFS), protéine C réactive (CRP), glycémie, ionogramme sanguin, urée, créatinine, taux de prothrombine (TP), International Normalized Ratio (INR), troponine T cardiaque (Tc). Durant l’évolution, les complications ont été répertoriées. Nos résultats ont été saisis et analysés par le logiciel d’étude statistique SPSS 20. Une description de l’ensemble de la population a été faite. Les résultats ont été exprimés par la moyenne ± écart-type. Les variables quantitatives ont été comparées par le test de Chi2 ou par le test de Fischer. Les variables qualitatives ont été comparées par le test de T de Student. Le risque d’erreur a été fixé à 0,05.

## Résultats

Nous avons inclus 200 patients durant une période de quatre mois. Les principales caractéristiques épidémio-cliniques sont présentées dans le Tableau 1. Cinquante-trois pourcent des patients ont été hospitalisés au service de neurologie (53%). Douze pourcent, ayant présenté une symptomatologie grave d’AVC, ont été hospitalisés au service des urgences et 3% dans d’autres services internes (4 patients en cardiologie, 2 patients en neurochirurgie). Trente-deux pour cent des patients ont été suivis en ambulatoire à la consultation externe de neurologie. Sur le plan biologique, une hyperglycémie a été constatée chez 103 cas (51,50%) dont 36 (34,95%) ne sont pas connus diabétiques. Une CRP élevée a été notée chez 95 patients dont 18 étaient fébriles à l’examen initial. La troponine Tc était positive chez 54/ 152 cas (35,5%) dont 2 ayant des signes électro cardiographiques d’infarctus du myocarde (IDM). Sur le plan radiologique, le scanner cérébral a été l’examen radiologique de première intention dans 98,5%. L’IRM cérébrale a été d’emblée réalisée en première intention chez 3 patients (une femme enceinte, un sujet jeune ayant eu un traumatisme cervical et un patient ayant eu une symptomatologie évocatrice d’une lésion du tronc cérébral). Les caractéristiques radiologiques sont détaillées dans le Tableau 2.

**Tableau 1:** Caractéristiques épidémio-cliniques de la population étudiée

**Age moyen (ans) ± écart-type**		68,45 ± 13,97
**Répartition des patients selon l’âge (n,%)**	**< 50 ans**	18(9%)
	**ans**	137(68,5%)
	**>80 ans**	45(22,5%)
**Sex-Ratio (H/F)**		1,59
**Patients vivants à Sfax (n,%)**		86(43%)
**Niveau socioéconomique bas (n,%)**		128(64%)
**Facteurs de risque vasculaire (n,%)**	**Sédentarité**	143(71,5%)
	**Hypertension artérielle**	124(62%)
	**Tabagisme**	89(44,5%)
	**Diabète**	67(33,5%)
	**Antécédent d’AVC**	40(20%)
**Délai entre la constatation des symptômes et l’arrivée au service des urgences (n, %)**	**< 1 heure**	40(20 %)
	**1-3 heures**	56(28 %)
	**>3heures**	104(52%)
**Score de Glasgow initial** **(moyenne ± écart-type)**		13,96±2,66
**NIHSS initial** **(moyenne ± écart-type)**		10,58±8,56
**MIF initial** **(moyenne ± écart-type)**		60±4,9
**Pression artérielle moyenne initiale (mmHg) ± écart-type**	**Systolique**	148,86±32,65
	**Diastolique**	83,57±16,87
	**Différentielle**	65,24±21,87
**Température > 38° (n,%)**		28(14%)

**Tableau 2:** Caractéristiques radiologiques de la population étudiée

Type d’AVC (n,%)	Ischémique: 159(79,5%)	Topographie sylvienne total: 24(15%)
	Hémorragique: 41(20,5%)	Hématome profond: 28(68,3%)
Signes scannographiques en cas d’AVCI (n,%)	Hypodensité franche	70(44%)
	Signes précoces	19(12%)
	Normal	70(44%)
Effet de masse sur la ligne médiane (n,%)		41(20,5%)
Engagement (n,%)		20(10%)
Œdème péri lésionnel (n,%)		20(10%)
Inondation ventriculaire (n,%)		12(6%)
Hydrocéphalie (n,%)		9(4,5%)
Hémorragie méningée (n,%)		6(3%)

**Figure 1:**
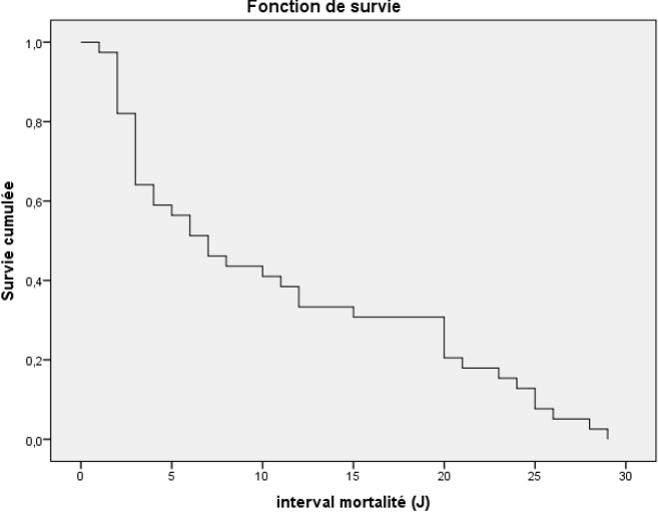
Courbe de survie Kaplan Meier de la population étudiée

En cas d’accident vasculaire cérébral ischémique (AVCI), un traitement par antiagrégant plaquettaire a été prescrit chez 130 patients (81,76%). Un traitement par anticoagulant a été prescrit chez 29 patients (18,23%). Un traitement antihypertenseur injectable à base d’inhibiteur calcique (nicardipine) a été prescrit chez 68,3% et 1,9% des patients atteints d’AVCH et ischémique respectivement. En cas d’accident vasculaire cérébral hémorragique (AVCH), une dérivation ventriculaire externe a été réalisée chez 22% des patients pour hydrocéphalie aiguë. Le mannitol a été prescrit chez 19,5% et 6,5% des patients atteints d’AVCH et ischémique respectivement et ayant des signes d’hypertension intracrânienne. Quinze pourcent des patients ont été intubés et ventilés. Plusieurs types de complications ont été notés à la phase aiguë de l’AVC. Les complications neurologiques ont été les plus fréquentes (58%), suivies par les complications infectieuses (24,3%) et métaboliques (15,5%). Après un mois de suivi, la mortalité a été de 19,9% avec un délai moyen de 10,28 ± 9,23 jours (Figure 1). Le MIF à J30 a varié de 18 à 126 avec une moyenne de 87,52±39,42. Une récupération fonctionnelle complète a été notée chez 29,8% des survivants. Les facteurs de mauvais pronostic vital à la phase aiguë sont détaillés dans le Tableau 3.

**Tableau 3:** Facteurs de mauvais pronostic vital

	Survivants (n=161)	Décédés (n=39)	P		Survivants (n=161)	Décédés (n=39)	P
Homme (n = 123)	93(57,77%)	30(77%)	0,027	Troponine (ng/l) (moyenne)	0,14	0,64	0,015
Tabagisme (n=89)	66(42%)	23(25,8%)	0,043	Urée (mmol/l) (moyenne)	6,42	11,7	<0,001
Antécédent d’AVC (n=40)	27 (16,77%)	13(33,33%)	0,02	La nature hémorragique de l’AVC (n=41)	27(16,77%)	14(35,89%)	0,008
Score de Glasgow initial (moyenne)	14,81	10,46	<0,001	Topographie sylvienne totale de l’ischémie (n=24)	9(5,6%)	15(38,46%)	<0,001
NIHSS initial (moyenne)	8,22	20,33	<0,001	Présence de signes précoces (n=19)	12(7,45%)	7(17,94%)	0,008
Céphalées (n=42)	29(18%)	13(33,33%)	0,035	Effet de masse (n=41)	16(9,93%)	25(64,10%)	<0,001
Crise épileptique (n=11)	5(3,10%)	6(15,38%)	0,003	Engagement (n=20)	1(0,62%)	19(48,71%)	<0,001
Signe de Babinski (n=65)	30(18,63%)	25(64,10%)	0,003	Hémorragie méningée (n=6)	1(0,62%)	5(12,82%)	0,018
Mydriase (n=9)	1(0,62%)	8(20,51%)	<0,001	Inondation ventriculaire (n=12)	0	12(30,76%)	<0,001
Aphasie (n=46)	35(21,73%)	11(28,20%)	<0,001	Hydrocéphalie (n=9)	0	9(23%)	<0,001
Déviation conjuguée de la tête et yeux (n=46)	22(13,66%)	24(61,53%)	<0,001	Œdème cérébral (n=20)	5(3,10%)	15(38,46%)	<0,001
Pression artérielle systolique moyenne (mm Hg)	144,52	166,79	<0,001	Assistance respiratoire (n=30)	1(0,62%)	29(74,35%)	<0,001
Pression artérielle Diastolique moyenne (mm Hg)	82,09	89,69	0,01	Traitement antiœdémateux (n=18)	10(6,21%)	8(20,51%)	0,005
Pression artérielle différentielle moyenne (mm Hg)	62,37	77,1	<0,001	Traitement antihypertenseur (n=31)	18(11,18%)	13(33,33%)	0,001
Fièvre (n=28)	12(7,45%)	16(41%)	<0,001	Transformation hémorragique (n=10)	0	10(25,64%)	<0,001
Glycémie (mmol/l) (moyenne)	8,06	11,07	0,001	Épilepsie vasculaire (n=22)	6(3,72%)	16(41%)	<0,001
CRP (mg/l) (moyenne)	20,73	50,95	0,002	Complications infectieuses (n=37)	16(9,93%)	21(53,84%)	<0,001
GB (éléments/mm^3^) (moyenne)	8884,52	11802,56	0,006	Complications métaboliques et/ou de l’hémostase (n=26)	15(9,31%)	11(28,20%)	0,002
Créatininémie (µmol/l) (moyenne)	95,147	145,79	0,035	Complications liés au décubitus (n=20)	12(7,45%)	8(20,51%)	0,015

## Discussion

Nous avons choisi de mener une étude prospective en définissant au préalable les données à recueillir afin d’assurer l’homogénéité des conditions de recueil. Nous avons répertorié, durant une période de 4 mois, les patients pour lesquels le diagnostic d’AVC a été retenu aux deux CHU de référence de la ville de Sfax. Ces patients ont eu un examen neurologique par le même clinicien afin de minimiser le biais de collection de données. D’autre part, pour une meilleure reproductibilité, nous avons utilisé des échelles de mesure fiables pour minimiser les biais de mesure. Mais, comme toute étude hospitalière, il existe des biais de sélection. En effet, on n’a pas pu recruter tous les patients d’AVC de la ville de Sfax ayant un AVC. En effet, certains patients n’ont pas consulté probablement du fait d’une symptomatologie mineure ou de signes non reconnus comme manifestations possibles d’AVC ou au contraire une évolution grave et qui meurent avant d’accéder l’hôpital. D’autres ont été pris en charge par des médecins de libres pratiques. Certains ont consulté un hôpital régional sans être adressés au service des urgences du CHU. Ainsi, les chiffres d’incidence et de prévalence n’ont pas pu être réalisés puisque notre série n’a pas été représentative de tous les cas d’AVC survenant dans la région de Sfax. De plus, elle a englobé des cas provenant d’autres gouvernorats. Toutefois, les résultats de notre étude peuvent être considérés comme une base de données pour apprécier l’ampleur de cette pathologie dans la région de Sfax et pour l’ajustement des attitudes de la prise en charge. Nous avons étudié par la suite les facteurs prédictifs du pronostic vital. Le taux de mortalité, à un mois dans notre série (19,5%), est semblable à celui rapporté dans les pays arabes et plus élevé que celui rapporté dans les pays développés [6, 7]. La mortalité dans les pays industrialisés est en continuelle baisse grâce aux nouvelles stratégies de prise en charge à court et à long terme (7). Ceci s’applique aussi à nos résultats, en effet la mortalité dans l’étude de Damak *et al*. menée à Sfax en 2004, a été de 27% à un mois [8]. Le taux de dépendance post AVC varie dans le monde de 18,5 à 72,5% [9]. Dans notre série, une récupération fonctionnelle complète (MIF=126) a été notée chez 29,8% des survivants. Ce taux est multiplié par 3 par rapport à celui de l’étude de Damak *et al*. (10%) [8]. Ceci pourrait être expliqué par une amélioration de la politique sanitaire sur la prévention primaire et secondaire de l’AVC dans notre région et une meilleure sensibilisation de notre population à l’urgence de la pathologie neuro-vasculaire et son retentissement fonctionnel.

*Sur le plan démographique*, l’âge avancé n’aggrave pas le pronostic vital à la phase aiguë dans notre étude. Cependant, d’après la littérature, il a un impact négatif quel que soit le type de l’AVC [10–12]. D’ailleurs, dans une analyse récente du programme américain « Get With The Guidelines-Stroke (GWTG) », il a été démontré que le risque de décès intra hospitalier chez les sujets âgés augmente pour chaque augmentation de l’âge de 10 ans [13]. Nos résultats pourraient être expliqués par un biais de recrutement. En effet, les sujets très âgés qui présentent une symptomatologie grave d’AVC sont généralement pris en charge à domicile par leur médecin traitant vue que la famille accepte facilement la fatalité de la pathologie. Le sexe masculin a été corrélé à un mauvais pronostic vital. Or, les données de la littérature sont contradictoires et la plupart des études rapportent que le sexe n’a pas d’influence sur la survie [10, 14]. Le NSE bas n’a pas été retenu comme un facteur de mauvais pronostic vital. L’influence du NSE sur la mortalité, prouvée dans la littérature, est d’autant plus importante qu’il est associé à un bas niveau d’éducation [15, 16]. Parmi les ATCD de facteurs de risque vasculaire, la consommation de tabac et l’antécédent d’AVC ont été corrélés à un mauvais pronostic vital. Dans la littérature, il a été démontré que le tabac est un facteur de mauvais pronostic vital indépendant [17]. Il est considéré comme l’un des facteurs prédictifs de récurrence après un premier AVC [18]. Les récidives d’AVC sont associées à des taux de mortalité et d’invalidité plus élevés ainsi qu’une augmentation des coûts après un AVC [7, 19].

*Sur le plan clinique,* le score de Glasgow bas a été corrélé à un mauvais pronostic vital. En effet, l’existence d’un score de Glasgow inférieur à 10 au cours des 24 premières heures est un paramètre indépendant de mauvais pronostic vital, dans la plupart des séries [20,21]. Dans notre étude, la sévérité initiale d’un AVC mesurée par le NIHSS demeure l’un des principaux prédicateurs de mortalité après un AVC, concordant avec la littérature [22,23]. Dans l’essai de TOAST, un point supplémentaire sur le score NIHS initial diminue la probabilité de survie à 7 jours de 24% et à 3 mois de 17% [24]. Un NIHSS ≥ 16 prédit une forte probabilité de décès ou d’invalidité grave, alors qu’un score ≤ 6 prédit une bonne récupération [25]. La présence de céphalées à la phase aiguë a été corrélée à un mauvais pronostic vital dans notre série. Elles sont plus associées aux patients atteints d’AVCH. Ces patients sont plus susceptibles d’avoir des signes méningés et un score de Glasgow plus bas [26]. Une étude récente a démontré que les patients atteints d’AVCH et qui ont présenté initialement des céphalées, auront un taux de mortalité plus important à 30 jours [27]. Par ailleurs, la présence de crise symptomatique aiguë (CSA) ou d’épilepsie vasculaire a une valeur prédictive confirmée [28,29]. En effet, dans notre série, leur présence a été liée à un mauvais pronostic vital. Dans une étude canadienne, la présence de CSA ou d’épilepsie post AVC s’accompagnait d’une augmentation du risque de décès très significatif à 1 mois (36,2%) comme à 1 an (48,6%), avec, en cas de survie, une plus grande dépendance et des difficultés professionnelles [30]. L’existence d’une pression artérielle élevée a été identifiée depuis longtemps comme étant responsable du plus grand nombre de décès par AVC, comme ça été démontré dans notre étude [19,31,32]. La mortalité augmente de 3,8% pour chaque 10 mm Hg au-dessus de 150 mm Hg et de 17,9% pour chaque 10 mm Hg en dessous de 150 mm Hg [33]. Des essais cliniques ont démontré que la réduction de la pression artérielle par l’utilisation de diurétiques et/ou d’inhibiteurs de l’enzyme de conversion réduit la mortalité par AVC ainsi que sa récurrence [18]. Cependant, l’HTA est bénéfique en cas d’AVCI car elle permet de maintenir la perfusion dans la zone de pénombre ischémique au tour de l’infarctus, évitant ainsi la lésion neuronale définitive et elle est donc traitée si elle dépasse 220/120 mm Hg. Un état subfébrile (37,5°C à 38,5°C) ou fébrile (> 38,5°C) à la phase aiguë de l’AVC sont associés à des volumes relativement importants d’infarctus, un taux de mortalité élevé, rejoignant nos résultats [18]. Dans l’étude de Wang *et al*. une augmentation de 1°C de la température d’admission prédit indépendamment une augmentation relative de 30% du risque de mortalité à long terme. Dans cette étude, ni l’hyperleucocytose, ni l’existence d’une infection documentée n’apparaissent liées à la mortalité [34].

*Sur le plan biologique,* la moyenne des chiffres glycémiques initiaux chez les patients décédés a été significativement élevée comparativement aux survivants. Même si l’hyperglycémie peut être le reflet d’infarctus plus volumineux, donc plus graves, il est désormais admis que l’hyperglycémie, qu’elle soit réactionnelle ou liée à un diabète, est corrélée de façon indépendante au mauvais pronostic vital. Ainsi, l’hyperglycémie multiplie le risque de décès à 30 jours par 3 chez des sujets non diabétiques, et le diabète par un facteur de 3,5 chez l’homme et de 5 chez la femme [35]. La neurotoxicité de l’hyperglycémie semble apparaitre pour un seuil bas (7 mmol/L) en favorisant la croissance de l’infarctus en altérant la viabilité de la zone de pénombre ischémique [36]. Ainsi, la correction de l’hyperglycémie en phase aiguë apparait comme une cible privilégiée pour améliorer le pronostic dans la prise en charge à la phase aiguë d’un AVC. D’autre part, une CRP augmentée à l’admission a été un facteur de mauvais pronostic vital dans notre série. Des chiffres élevés de la CRP dans les 12 à 72 heures en post AVC ont été associés à un risque accru de décès qui est multiplié par 2 [37]. La relation entre l’augmentation de la CRP et de la mortalité post-AVC peut refléter en partie le dysfonctionnement des cellules endothéliales induit par l’inflammation et l’activation plaquettaire [38]. L’augmentation de la troponine Tc a été un facteur de risque de mortalité dans notre série à la phase aiguë de l’AVC. L’élévation de la troponine semble être un marqueur prédictif de mauvais pronostic vital que ce soit à court ou moyen terme, et ceci même en l’absence d’infarctus du myocarde et d’insuffisance rénale, à la fois à la phase aiguë de l’AVC et après un an [39–41]. Une des explications pourrait être la présence d’IDM silencieux ou non dans les semaines précédant l’AVC. Toutefois, l’augmentation de la troponine n’est pas liée uniquement à une ischémie myocardique associée mais s’intègre dans un mauvais état cardiovasculaire global. Elle représente un facteur de mauvais pronostic à court et moyen terme en étant le reflet de pathologies intercurrentes chroniques plus d’apparition de troubles cardiaques aigus [42].

*Sur le plan radiologique,* la nature hémorragique de l’AVC a été un facteur de mauvais pronostic vital concordant avec la littérature. En effet, dans l’étude Copenhagen, le taux de mortalité a été estimé entre 10,9% et 26,0% et entre 28,9% et 61,0% pour les patients victime d’AVCI et hémorragique respectivement et ceci entre J28 et J30 post AVC [14]. Un taux de survie plus bas au décours d’un AVCH a été expliqué par les coups plus graves survenant lors ce type d’AVC. De même, dans notre série, les signes radiologiques associés (œdème cérébral, effet de masse, inondation ventriculaire) étaient d’un grand apport pronostique et intérêt thérapeutique à la phase aiguë, quel que soit le type de l’AVC [43–46]. La dernière méta-analyse de la Cochrane Data base, comparant l’hospitalisation en UNV à celle d’un service de soins classique, montre que la prise en charge en UNV permet de réduire la mortalité des patients à 1 an de 14%. La quasi-totalité de cette diminution est obtenue dans les 30 premiers jours [47]. Ainsi, il serait urgent que le tunisien bénéficie d’une prise en charge précoce multidisciplinaire au sein d’une UNV adaptée, au moins dans tous les hôpitaux universitaires, puisqu’il est prouvé qu’elle améliore nettement le pronostic vital et réduit le coût direct de la prise en charge des AVC.

## Conclusion

L’AVC est une urgence diagnostique et thérapeutique. En termes de santé publique, l’AVC représente un véritable fléau sur le plan médical, social et économique. Les pays en voie de développement dont la Tunisie sont particulièrement affectés par l’impact de cette pathologie. Il serait urgent que le tunisien bénéficie de structure spécialisée au moins dans tous les hôpitaux universitaires compte tenu de l’espérance de vie qui ne cesse de s’allonger. En attendant l’ouverture d’une UNV dans notre institution, il semble important d’améliorer et d’harmoniser rapidement la filière de prise en charge des AVC à la phase pré et intra hospitalière. Ainsi, le pronostic vital des patients pourrait être nettement amélioré.

### Etat des connaissances actuelles sur le sujet

- L’AVC constitue un problème majeur de santé publique, tant par le nombre de personnes atteintes, en constante augmentation compte tenu du vieillissement de la population, que par ses conséquences médicales, sociales et économiques qui en découlent;
- Plus de 65% de la mortalité par AVC dans le monde est enregistrée dans les pays en voie de développement
- Une connaissance complète de l’impact des facteurs pronostiques sur l’évolution de l’AVC reste encore difficile à établir dans la région nord-africaine.

### Contribution de notre étude à la connaissance

- Nous avons mené ce travail prospectif pour dégager les facteurs prédictifs du pronostic vital à la phase aiguë de l’AVC artériel propres à une population du sud tunisien;
- Améliorer les stratégies de prise en charge des patients victimes d’AVC en créant une UNV dans la région de Sfax;
- Envisager une étude comparative afin de mesurer l’impact des mesures correctives.

## Conflits d’intérêts

Les auteurs ne déclarent aucun conflit d’intérêts.
